# Strategy for Non-Orthogonal Multiple Access and Performance in 5G and 6G Networks

**DOI:** 10.3390/s23031705

**Published:** 2023-02-03

**Authors:** Omer Mohammed Khodayer Al-Dulaimi, Aymen Mohammed Khodayer Al-Dulaimi, Maiduc Osiceanu Alexandra, Mohammed Khodayer Hassan Al-Dulaimi

**Affiliations:** 1 Department of Telecommunication Engineering, Polytechnic University of Bucharest, 060042 Bucharest, Romania; 2 Technical Communication Engineering, Al-Farahidi University College, Baghdad 10022, Iraq; 3 Department of Director for Management of Scientific Research, Polytechnic University of Bucharest, 060042 Bucharest, Romania; 4 Department of Computer Engineering, Al-Rafidain University College, Baghdad 10022, Iraq

**Keywords:** NOMA technology, 3GPP standardization, 5G systems, code division, LDS–CDMA, SCMA, PDMA, OFDMA

## Abstract

The purpose of this paper is to provide a high-level overview of the most important non-orthogonal multiple access (NOMA) protocols in 5G and 6G networks that incorporate code division within the context of 3GPP standardization. The article’s objective is also to look into and compare the various strategies that have been proposed as a solution to the issue of resource distribution to achieve high performance. Many different NOMA plans for 5G and 6G systems have been suggested by a multitude of businesses. NOMA is currently developing in two primary directions: one of them is with power division, and the other is with code division. During the process of standardization carried out by the 3GPP, the attention of the developers was concentrated in the second direction for the application of NOMA schemes in 5G and 6G systems. Hardware communication, also known as D2D communication, performs a significant role in the process of communication between devices. This will increase the efficiency with which network resources are utilized. Devices are now able to interact directly with one another, avoiding the need for transmission nodes. It also serves as one of the approaches to the problem of limited network coverage, which can be improved by utilizing D2D, and as a result fees and energy can be reduced. Increasing the size of the network is one way to achieve this goal, the explained of NOMA technology as well as its primary benefits in wireless technology. The most common variants of code division NOMA and the characteristics of those variants are discussed, as well as the opportunities and challenges associated with implementing those variants. NOMA protocols allow continuous expansion of wireless communication networks, i.e., 5G and 6G, which leads to enhanced performance of the networks.

## 1. Introduction

The development of multiple access technologies was a significant factor in the evolution of wireless communication [1]. The possibilities of technologies known as orthogonal multiple access technologies, in which the same resource (time or frequency) can be consumed by only one subscriber at a time, are severely restricted [2]. Non-orthognoal multiple access, or NOMA, protocols, which allow for the sharing of a single resource among a number of different subscribers, are a possible solution for the continuous expansion of wireless communication networks (5G, 6G) [3]. There are many variations of the NOMA technology that may be purchased from a variety of manufacturers and are described in a wide range of publications [4,5]. In this manuscript, the most widely used NOMA schemes with code separation, which have been suggested as part of the 3GPP simplification for 5G systems, are investigated; along with their primary benefits and drawbacks, as well as the challenges that prevent the implementation of NOMA systems [5].

## 2. Objective of the NOMA Conception

The spectrum and energy efficiency of wireless communication networks are greatly influenced by the use of various access strategies. Non-orthogonal multiple access (NOMA) are technologies that can increase the effectiveness of orthogonal access systems due to their compatibility with other multiple access methods, their adaptability in the use of system resources, and the simplicity with which they can connect and operate a large number of subscriber devices. Time division multiple access (TDMA) and frequency division multiple access (FDMA) are two well-established orthogonal signal separation technologies [5] employed in modern multichannel communication systems. When using orthogonal access, each user is given exclusive use of a separate signal or frequency range. Multiple users can share the same frequency or specific time in a network that employs non-orthogonal multiple access (NOMA). Furthermore, a NOMA system allows for flexible power distribution strategies to ensure a more equitable recycling of resources for users located further from the networks’ epicenter. It is important to recognize that the rapid evolution of today’s wireless communication networks necessitates a revaluation of traditional methods for creating such systems [5]. A rise in the number of subscribers is a higher priority in 5G and 6G wireless communication networks than it was in earlier generations, where an increase in the data transfer rate per subscriber was necessary. In 5G networks, NOMA technology was recommended particularly for mass connection of subscriber devices (IoT) where a high data transfer rate is also not essential for all users. When resources in the frequency and time domains are scarce, as they often are in orthogonal access systems, NOMA technology provides an efficient workaround [5].

**Figure 1:**
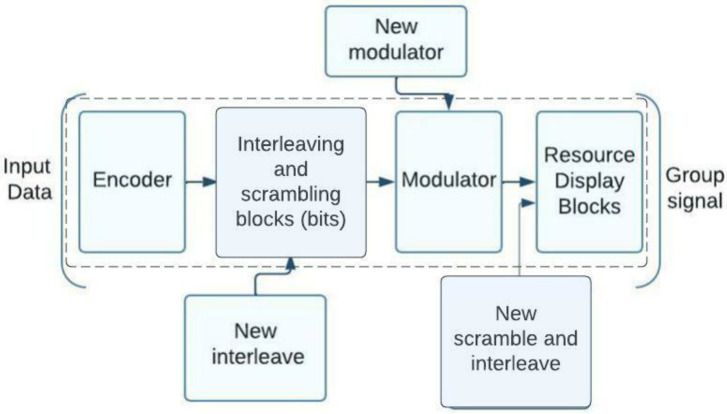
NOMA system transmitting side structure based on 5G standard.

NOMA standardization described in the 3GPP report is a measuring technique that was produced by the 3GPP working group for the NR (New Radio) standard for 5G systems [6]. It can be include many Technologies such as NOMA with pattern division (PDMA), resources spreading multiple accesses (RSMA), multi-user shared access (MUSA), multiple accesses Welch spreading multiple accesses (WSMA), and interleave-division multiple-access (IDMA). To generate NOMA signals for the uplink, the 3GPP document [7] suggests modifying the transmitting side of the NR 5G system. The Figure 1 shows the modified transmitting side of the NOMA system based on the 5G system’s standard framework. 3GPP specifications do not represent new digital processing blocks. Current standards must be modified to add new blocks to the 5G specification. The dotted lines indicate 5G processing blocks [7]. To implement NOMA, the 5G specification must include ways for delivering subscriber group signals. Developers and equipment manufacturers choose receiver structures. Group signals can isolate non-orthogonal resources of different users and boost individual user transfer rates. Additional processing at the character, bit, or both levels is used in code-separated NOMA systems. The structural scheme in Figure 1 shows how subscribers’ code sequences are created using coding, interleaving, and scrambling technologies.

As shown in Figure 1, the different types of NOMA employed in 5G systems necessitate either a redesigned modulator, a new scrambling block, a new six-block interleaving, or some combination of these blocks. It describes using specific NOMA schemes , how the algorithms for producing non-orthogonal multiple access group signals on the transmitting side can result in a wide variety of new block combinations in the 3GPP NOMA schemes. The 3GPP standardization process studied the advantages of NOMA and ultimately decided to add NOMA and orthogonal access for uplinks [8] to the 5G standard. Although NOMA has been shown to outperform current orthogonal multiple access technologies, they do so at the expense of complexity in the algorithms necessary to receive user signals [9]. When there are too many subscribers for the number of orthogonal cycles, NOMA technology comes into its own. If carriers are employed as the signal’s origination point, as they would be in an OFDMA scheme, then a discrete Fourier transform block must be placed after the modulator. When comparing NOMA systems with code separation (WSMA scheme) to multi-user MIMO systems, it was reported that the gain in noise immunity is not very substantial for 5G situations. However, a NOMA receiver has a significantly higher complexity, and algorithms with a manageable level of complexity are required for its implementation.

## 3. NOMA Code-Separated Techniques

Based on subscriber signal code division, 3GPP described various NOMA systems [9]. The proposed schemes for existing 5G scenarios did not show a significant advantage over the used 5G technologies; hence NOMA was not included in 5G standards but was further studied for new scenarios [9].

### 3.1. Development of NOMA Technology

Power division (power domain NOMA) and code division (code domain NOMA) are the two basic kinds of NOMA schemes. In the first case, channel users may broadcast at different decibel levels. Code division NOMA does not use orthogonal code sequences to partition subscriber signals, unlike traditional CDMA systems, which have a capacity proportionate to the number of code sequences. Successively adopting non-orthogonal codes increases the number of subscribers, but it requires expensive algorithms for multi-user reception. A network’s NOMA code division system divides shared time and frequency resources by subscriber code sequence. In contrast to CDMA, non-orthogonal sequences with a low correlation coefficient and many zero elements are used to separate subscribers [10].

ANOMA system has a far higher subscriber-to-signal base ratio than a traditional CDMA system. NOMA technology with code division includes orthogonal frequency division multiple access (OFDMA), code division multiple access (CDMA) with low density spreading (LDS), and sparse code multiple access (SCMA). LDS–CDMA uses a lower-density code sequence than CDMA. The information symbols it sends are superimposed on LDS sequences before transport on orthogonal OFDM carriers in low-density OFDM (LDS–OFDM) [10]. By broadcasting more symbols than orthogonal carriers, the communication system’s spectral efficiency can be enhanced compared to a typical OFDM system. Huawei’s sparse code sequence algorithm (SCMA) improved LDS–CDMA [10]. Unlike LDS–CDMA, SCMA directly converts information bits to sparse code words using sophisticated code sequences. We can expect better characteristics by increasing receiving end processing complexity. The lattice partitions multiple access (LPMA) system, which uses lattice codes, is another NOMA-related multiple access approach [10].

Other promising NOMA systems are SDMA and PDMA. To prevent cross-user interference, PDMA systems multiplex and transmit subscriber data streams using non-orthogonal templates. PDMA can be used with other multiple-access methods since it can multiplex in both the spatial and coding domains [10]. Using a software-configurable ad hoc interface for 5G systems, code-separated NOMA can be implemented [11]. This idea ensures 5G network modes and services are supported by diverse access strategies. In the Internet of things mode, shared multiple access (MUSA) schemes enable mass subscriber device connectivity in 5G systems [12]. The idea of a software-configurable radio interface for 5G systems was proposed to accommodate the many modes and services of 5G networks, such as the various types of NOMA with code division detailed below [13]. 5G networks must use multiple access joint (MUSA) technologies to connect subscriber devices in the Internet of things mode [13].

### 3.2. Low-Density Spreading by CDMA Technology

LDS sequences, with CDMA and OFDMA were used to create the first code division NOMA schemes [14]. LDS–CDMA, an enhancement of CDMA employing low-density codes, was introduced over ten years ago [15]. The LDS–CDMA technology uses LDS sequences to apply a multi-user reception method, which is close to the principal-component-criterion optimal algorithm [16]. Due to LDS sequences, LDS–CDMA technology can use a multi-user reception algorithm that is comparable to the algorithm that maximizes probability of users [17] ]. As users multiply in a typical CDMA system, reciprocal interference increases. To reduce noise, orthogonal codes with a changeable base were proposed, but such a system is not meant to work in overload mode, i.e., when the number of subscribers greatly exceeds the number of viable orthogonal code sequences. LDS–CDMA uses low-density coding sequences with many zero elements. Such LDS sequences reduce subscriber signal interference and allow mass connection and overload mode operation. LDS–LDS CDMA’s code format simplifies user-signal processing compared to CDMA. Consider an LDS–CDMA system with *K* = 6 users using sequences of length *N* = 4. Information symbols reflect into code sequences using the following formula:

(1) $$S=\left[\begin{array}{c} \begin{array}{cc} 1 & \begin{array}{cc} 1 & \begin{array}{cc} 1 & \begin{array}{cc} 0 & \begin{array}{cc} 0 & 0 \end{array} \end{array} \end{array} \end{array} \end{array}\\ \begin{array}{cc} 1 & \begin{array}{cc} 0 & \begin{array}{cc} 0 & \begin{array}{cc} 1 & \begin{array}{cc} 1 & 0 \end{array} \end{array} \end{array} \end{array} \end{array}\\ \begin{array}{c} \begin{array}{cc} 0 & \begin{array}{cc} 1 & \begin{array}{cc} 0 & \begin{array}{cc} 1 & \begin{array}{cc} 0 & 1 \end{array} \end{array} \end{array} \end{array} \end{array}\\ \begin{array}{cc} 0 & \begin{array}{cc} 0 & \begin{array}{cc} 1 & \begin{array}{cc} 0 & \begin{array}{cc} 1 & 1 \end{array} \end{array} \end{array} \end{array} \end{array} \end{array} \end{array}\right]\;M^{k}M^{w}$$

**Figure 2:**
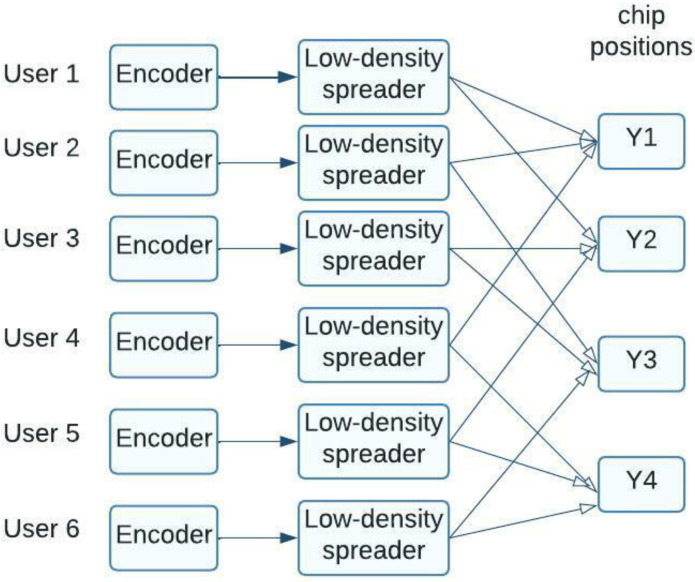
Illustration of an LDS–CDMA system’s basic architecture.

In this case, the signal base is represented by the number of rows in the matrix *S*, and the number of subscribers who access these sequences is represented by the number of columns. With a loading ratio of 150%, the system can support far more users than standard CDMA networks. A huge number of zero elements characterize the structure of the matrix *S*. A graphical representation of this system is provided in Figure 2; $x_{i}$ represents subscriber information symbols and $y_{i}$ represents elements in received code sequences.

The MPA algorithm (message passing algorithm) can repair mistakes on the receiving side of the NOMA multi-user demodulation system. The MPA algorithm simplifies a demodulator using sequential interference suppression and employs a graph format to show informational symbols as a resource for demodulation. Errors can be fixed with a turbo decoder. These iterative processing approaches can improve demodulation quality (but increase processing complexity). NOMA systems with LDS code sequences make multi-user demodulators, especially sequential interference suppression-based ones, easier to build. Low density spreading CDMA sequences reduce user-signal interference even more than regular CDMA systems. CDMA and NOMA systems with code separation have the following reception processing computational complexity. The receiver complexity in a typical CDMA system (optimal by maximum likelihood criterion) is $M^{k}$*,* where K is the number of active users and M is the modulation multiplicity. This can be used to evaluate the computational difficulty of processing at the receiver for CDMA and NOMA systems with code division. The receiver’s complexity is $M^{w}$, *W < K* if the NOMA system uses *W* code sequences, where K is the number of active users and M is the modulation multiplicity. If the NOMA system uses W code sequences, complexity of an LDS-computational OFDM’s receiver system is larger than of OFDMA due to LDS sequences.

## 4. Code Division with Processing

**Figure 3:**
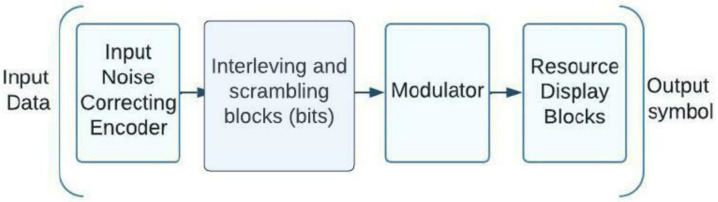
A schematic of the sending side of a NOMA system that shows processing at the bit level.

In the development of 3GPP standardization, two primary groups of schemas were presented for the development of the NOMA direction with code division. These groups are distinguished by the type of processing they perform: either at the bit level (before the modulator) or at the symbol level (after the modulator). Let us investigate these NOMA variants with code division in greater depth, focusing on their structural schemes that illustrate transmission processing and the most common approaches to producing code sequences. Scrambling and interleaving at bit level: The randomization of NOMA subscriber signals at the bit level makes separation possible, and the 3GPP document [18], takes two methods of randomization, scrambling and interleaving into account to facilitate this separation. Figure 3 is a block schematic of a bit-level processing NOMA system. An encoder that can survive noise is supplied with user input; this is followed by bit-level scrambling or interleaving, modulation, and symbol-level mapping of the received information into the available resources. As an illustration, subcarriers can be employed as sources, followed by a discrete Fourier transform, as discussed in the 3GPP report [18]. The modulator calls for a block of discrete Fourier transform once the resources are spent, such as subcarriers (DFT).

Several bit-scrambling NOMA methods, such as LG Electronics’ NCMA (network-coded multiple access) and Intel’s LCRS (low code rate spreading), have been proposed for 5G standardization. A NOMA system can use the scrambling process described in the 3GPP specifications [19]. A NOMA system uses subscriber-specific interleaving patterns for bit-level interleaving. Samsung’s IGMA, Wireless LLC’s IDMA, and Intel’s LCRS are illustrations of such NOMA schemes [19]. It should be emphasized that NOMA requires new interleaving blocks in a standard 3GPP structure, implemented based on the standard low-density block coding (LDPC), and included in the 5G specifications by adding distinct cyclic shifts to segregate signals from different subscribers [19]. IDMA’s unique code sequences separate subscriber signals, which is more intriguing. Thus, IDMA is a CDMA with shifting code sequence chips, and by adding a shifting block on the sending side, an iterative multi-user reception method may recover the sequence.

## 5. MA with Code Separation

The 3GPP document also suggests NOMA circuits with processing at the symbol level (after the modulator), which include numerous variations that differ in the way NOMA group signals are produced. These NOMA circuits feature processing at the symbol level [19]. When processed at the symbol level, group signals that are compliant with the 5G requirements can be generated using a number of different methods [20], such as the individual interleaving for each subscriber with the addition of invalid characters or the use of individual code sequences for diverse subscribers using modulation types that are already specified in the 5G standards.

### 5.1. Individual Code Sequences for Traditional 5G Modulation Subscribers

**Figure 4:**
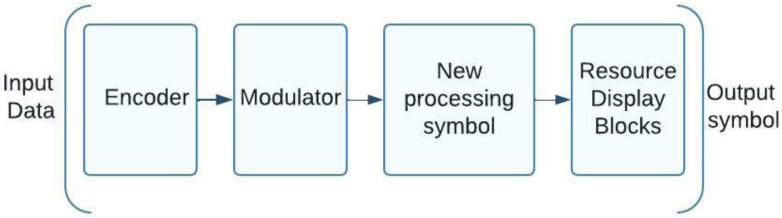
A schematic of a 5G-modulated NOMA transmission system.

Code sequences with a high number of zero elements and a low level of cross-correlation between the signals of various subscribers are frequently used for this method of group signal formation [21]. The building blocks of these sequences can be derived from the 3GPP-defined BPSK, QPSK, or QAM signal structure standards [22]. Figure 4 shows the transmitting side structure of a NOMA system using the 5G modulation that has been in use up to this point. One of the processing bits can be performed at the higher level bits after noise-resistant coding of the input data stream has been carried out. In the final step, traditional 5G modulation is used to process the data at the character level. The production of group signals using this method is characterized by a low level of mutual correlation between the signals of various subscribers and by the employment of code sequences with a large number of zero elements [23]. Bit-level processing can begin with noise-resistant coding of the incoming data stream or any of the aforementioned techniques. Once the modulation is complete, symbol level processing in 5G legacy mode is executed.

For the 3GPP standardization, different organizations have suggested distinct NOMA code sequence architectures for character processing [24]. The Welch and Grassmannian sequences underlie the Ericsson (WSMA), and Nokia (NOCA) schemes, respectively, whereas the generalized Belch sequences method underlie the NTT DOCOMO (UGMA) system. Qualcomm’s RSMA scheme, which implements a character-level scrambling technique, is frequently cited as the system used in publications.

### 5.2. Implementing Belch Sequences in NOMA Models

Two examples of Welch sequence (WBE, Welch bound equality)-based NOMA variants are Qualcomm’s RSMA and Ericsson’s WSMA. Welch boundary equality is an equality used to create WBE sequences.

(2) BWelch=K2N

**Figure 5:**
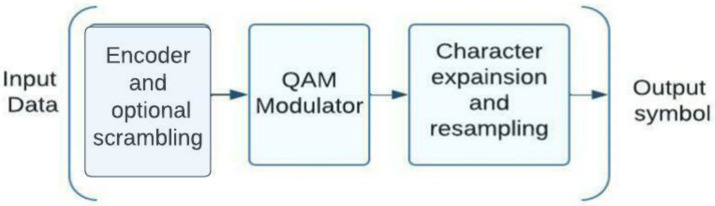
Aspect of the WSMA scheme concerned with structural transmission.

The 3GPP technical report [24] provides several examples of how to generate WBE sequences. Co-boundary of the cross-correlation of any of the *K* sequences of length *N* is equal to (2). The block diagram of the WSMA system’s subscriber station’s transmitter is depicted in Figure 5. It is optional to scramble the bits after they have been subjected to noise-correcting coding (bit-level processing). The data are then subjected to quadrature amplitude modulation (QAM), spread, and interleaved with code sequences. The WSMA system employs short WBE sequences with a low cross-correlation coefficient. Meanwhile, unlike the SCMA method, the WSMA scheme does not include sparse sequences [25].

The WSMA technique uses symbol-level signal formation, with individual vectors (sequences) of length *L* predetermined and assigned to individual subscribers. There are correlational features of these vectors. We will take a closer look at the WSMA scheme with *K* subscribers, specifically at the structure and features of the matrix of WBE sequence. Let there be K vectors of sequences $S_{k}.k=1,\ldots ,K,$ denoting user code sequences for which $\left\|S_{k}\right\|=\sum_{l=1}^{L}S_{{k.l}^{2}}=1,$ then the matrix of WBE sequences has the structure $S=[S_{1}.S_{2},.\ldots .{,S}_{K}]$ and size L × K. The load factor of the WSMA system is determined by the K/L ratio; moreover, to support a large number of subscribers, it is necessary that (K/L)>1. Depending on the required characteristics of the sequence matrix S, the efficiency indicator is selected, for the WSMA scheme, total square correlation (TSC) coefficient, which is bounded by equality (2) and specified by the relation

(3) $$TSC=\sum_{i=1}^{K}\sum_{j=1}^{K}{\left|\begin{array}{cc} S_{i} & {\prime S}_{j} \end{array}\right|}^{2}\geq K^{2}/L$$

where the transposition and complex conjugation of elements of the vector $S_{i}$. When this relation is satisfied, the set sequence S corresponds to the Belch boundary; however, the vectors of this set satisfy the Belch boundary not individually, but collectively. Thus, multiple sets *S* exist that correspond to the given performance metric and also satisfy the Welch boundary. This scheme’s appeal is NOMA implementation untruths. In fact, its optimization guarantees a low correlation between vectors in the set *S* and optimizes the WSMA system capacity. Here, we see a sample of such a set *S* of WBE sequences (WSMA codebook) taken from the 3GPP publication [26]. Up to four active subscribers (*K* = 4 sequences) of length *L* = 8 per one orthogonal resource are supported in a WSMA system with this matrix *S*. With *K/L* = 2, the system operates at a load of 200%. Each codebook column corresponds to a subscriber and is represented by a complex WBE sequence with a unit norm $\mu ={max}_{i\neq j}P_{ij}$. Where $P_{ij}$ the cross-correlation coefficients are potentially useful for creating WBE sequences. $P_{ij}$ It’s mentioned in locations we may find the correlation coefficients between variables. The Grassmann set is a collection of such WBE sequences, and the packing problem is the optimization issue [27]. It is worth noting that subsets of WBE sequences with various correlation qualities are created by independent optimization of each efficiency indicator. Optimization of the parameter (3) yields WBE sequences with varying degrees of cross-correlation, while optimization of the parameter WBE yields sequences with a constant degree of cross-correlation [28]. As illustrated in Table 1.

Optimization of the chord distance $d_{cord}$ makes it possible to obtain a level of mutual correlation between several code sequences close to zero, while the optimization task is to pack the subspace *G* [29]. The x-Nordic distance between two sequences is determined by the formula

(4) $$d_{cord}={min}_{G}\left({max}_{1\leq i\leq j\leq k}\sqrt{{1-\left|S_{i}^{\prime }\cdot S_{j}\right|}^{2}}\right)$$

Table 2 explained various changes of the Gram|S’S| that demonstrate the correlation features of WBE-sequences for the WSMA system. For a system with *K* = 4 active subscribers per one orthogonal resource, S sets of these sequences were constructed by optimizing different indicators for the situation *K* = L with L-length WBE-sequences (100% utilization). In this way, the following structure of Gram determinants is obtained for various values of the optimal parameters of TSC and *d_cord_*:

### 5.3. Grassmannian-Sequence-Based NOMA Protocols Founded on the Generalized Welch Equality

Grassmannian sequences provide the foundation for LG Electronics’ NCMA proposal, which makes use of these sequences. When creating these kinds of sequences, a more severe optimization strategy is applied in comparison to the WSMA scheme. This strategy involves minimizing the greatest level of mutual correlation that can exist between any two WBE sequences. A linear space-packing problem characterizes the process of generating Grassmannian sequences. It is possible to define the set of Grassmannian sequences as follows:

(5) $$\sum_{I=1}^{K}\sum_{J=1}^{K}P_{I}P_{J}{\left|S_{i}S_{j}\right|}^{2}\geq {\left(\sum_{k=1}^{K}P_{k}\right)}^{2}/N.$$

(6) $$R_{s}={\left\|S^{\prime }PS\right\|}^{2}=\sum_{i=1}^{k}\sum_{j=1}^{k}P_{i}P_{j}{\left|S_{i}{\prime S}_{j}\right|}^{2}$$

The matrix is $G=\left[S_{1}\ldots .S_{K}\right],$ which consists of vectors of sequential values and $G\subset C^{N\times K}$ where $C^{N\times K}$ is the set of complex matrices with the dimensions N×K, *N* is the length of the sequences, and *K* is the total number of sequences. The NTT DOCOMO Company’s UGMA system utilizes generalized Welch-bound equality (GWBE) sequences in order to division subscriber signals [30]. The GWBE is the foundation for these sequences, in contrast to the WBE sequences that correspond to the Welch bound (2). To the Belch border, this encompasses the following components in the total power $P_{k}$ of signals K of subscribers:

The GWBE sequences that were just described are made up of complex numbers, the real and imaginary components can be quantized utilizing discrete value ranges. ZTE’s MUSA method [31] likewise makes use of sequences that contain elements that have been quantized. The QPSK and QAM signal constellations can provide the building blocks for the code sequences, which can then be used. For instance, the number of such sequences is equal to times the length of the sequences $4^{N}$ in the case of QPSK, where *N* is the length of the sequences. The 3GPP document has other examples of sequences. These sequences are created using a computer optimization process to establish a low mutual correlation between sequences with varying cyclic shifts. The NOCA scheme from Nokia also employs quantized sequences using elements from the QPSK signal constellation.

### 5.4. Sequence-Based NOMA Schemes with Reconfigured Modulation Based on Sparse Templates

**Figure 6:**
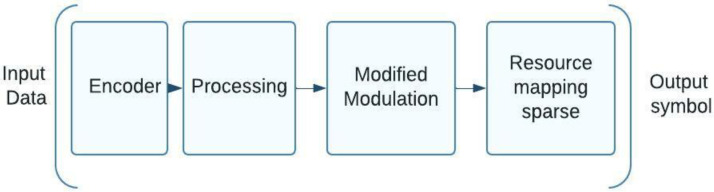
Sparse pattern and modified 5G modulation NOMA system structure.

Figure 6 illustrates a block diagram of a NOMA system with sparse patterns and modified 5G modulation on the transmitting side of subscriber stations. Following sparse bit-level encoding and processing, it is mapped to information symbols using a customized technique (modified modulation). The algorithm provided in the paper [32] is then applied to the resources based on particular sparse templates, displaying the sequence of symbols obtained at the modulator’s output. Thus, the method depicted in Figure 6 calls for processing on the transmitting side to make use of new types of modulation and expansion using sparse patterns to create that sparse character sequence. Huawei’s planned SCMA scheme is an example of a NOMA of this kind [33]. Sparse signals are produced by adding zeros to the code sequences used to construct group signals of subscribers in the SCMA scheme using special templates. In this scenario, SCMA employs codes where the number of leading zeros is constant across all templates. For the purpose of subscribers, below is a sample sequence template with four elements:

(7) $$S=\left[\begin{array}{c} \begin{array}{cc} 1 & \begin{array}{cc} 1 & \begin{array}{cc} 1 & \begin{array}{cc} 0 & \begin{array}{cc} 0 & 0 \end{array} \end{array} \end{array} \end{array} \end{array}\\ \begin{array}{cc} 1 & \begin{array}{cc} 0 & \begin{array}{cc} 0 & \begin{array}{cc} 1 & \begin{array}{cc} 1 & 0 \end{array} \end{array} \end{array} \end{array} \end{array}\\ \begin{array}{c} \begin{array}{cc} 0 & \begin{array}{cc} 1 & \begin{array}{cc} 0 & \begin{array}{cc} 1 & \begin{array}{cc} 0 & 1 \end{array} \end{array} \end{array} \end{array} \end{array}\\ \begin{array}{cc} 0 & \begin{array}{cc} 0 & \begin{array}{cc} 1 & \begin{array}{cc} 0 & \begin{array}{cc} 1 & 1 \end{array} \end{array} \end{array} \end{array} \end{array} \end{array} \end{array}\right]$$

In this configuration, each column of the matrix *S* corresponds to a different subscriber, and the number of rows is equal to the total number of elements in each sequence. The SCMA system load factor for the seventh template is *K/N* = 6/4, which equals 1.5 (load 150%). In comparison to the SCMA scheme, the improved PDMA method developed by CATT does not have a set limit on the number of elements that are either zero or non-zero [34]. The NOMA schemes allow for the selection of sequence members from more complicated sets, such as $\left\{0,1,-1,j,-j\right\}$. In this particular instance, the input stream consisting of M bits is shown in sequence with the assistance of a unique transformation matrix that has the dimension $N\times 2^{M}$, 

### 5.5. A Character-Level Extension and Scrambling Implementation of a NOMA System

**Figure 7:**
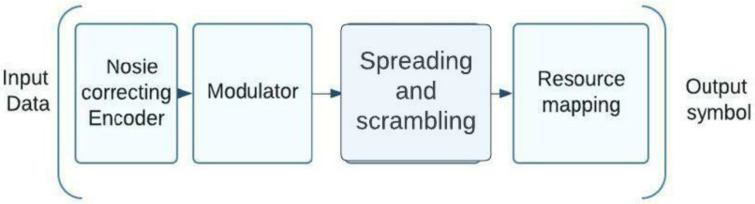
Design of a NOMA system’s character-level hybrid processing.

Figure 7 illustrates that the process for scrambling can also be used at the symbol level. This is shown in the diagram. In this particular variation of NOMA, the so-called hybrid symbol processing is carried out at the output modulator. This involves expansion as well as scrambling. When utilizing the OFDMA technology, following the display of the cycles, a discrete Fourier transform block is required. Bit-level processing may also be applied as an additional processing option [35].

In the RSMA technique introduced by Qualcomm, each modulated symbol has its own brief expanding sequence that is employed during symbol expansion. And in the RSMA plan for creating group signals, both short and long code sequences are utilized sequences (scrambling at the character level). It is possible to use Zadoff Chu sequences as a basis to develop new scrambling sequences.

### 5.6. Character-Level NOMA Scheme with Zero-Adding Interleaving

**Figure 8:**
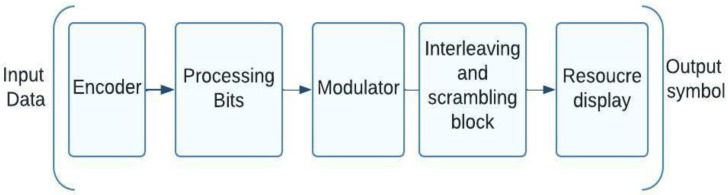
NOMA system structure with interleaving and zero elements.

Figure 8 illustrates a block diagram of a NOMA system that includes interleaving and zero element addition at the symbol level (from the perspective of the subscriber station). After the interference code has been processed, the bit level is entered. The Samsung IGMA (interleaved mapping multiple accesses) system employs a similar transmitting side arrangement [36].

When data are processed at the character level in the IGMA scheme, a specialized display process is carried out. During this process, character sequences are placed through the insertion of null components and elements. As a consequence of this, sparse sequences of characters are produced, and the degree of sparseness can be adjusted by manipulating the display matrix [37].

## 6. Characteristics of the Methodology of Implementing NOMA Schemes

All of the suggested NOMA variants come with their own set of benefits and challenges. All of these connections are designed for use in the uplink, or between subscriber terminals and the base station. Since NOMA systems use complicated code sequences, sophisticated methods of modulation and coding, and complex algorithms for multi-user reception of such signals [38], their processing is inherently difficult. The widely used SCMA method, for instance, requires both sophisticated transmitting code books and elaborate receiving signal processing algorithms. Complicated algorithms for the multi-user receipt of such signals are necessary for the MUSA system due to its usage of complex sequences. Trellis coding techniques are the most difficult to implement in an LPMA setting. Research into efficient algorithms for creating code books and coding, innovative methods of modulation and mapping, and combinations with orthogonal access schemes and MIMO technology are all areas that 3GPP standardization members have identified as requiring further investigation [39]. The 3GPP paper emphasizes the significance of developing low-computability algorithms for multi-user receipt of NOMA signals [40].

## 7. Conclusions

As more and more subscribers connect their devices to wireless networks in the present and the future, the industry of wireless communication systems is faced with a growing challenge: how to maximize spectral efficiency and throughput. Consequently, users have high expectations for the new networks in terms of the energy economy, communication reliability, and data transmission rates. It was suggested that non-orthogonal multiple-access technology be used to address these issues (NOMA). Recently, a significant amount of effort has been into studying how to make 5G networks more efficient by incorporating NOMA technology. Many different types of non-orthogonal access (NOMA) have been proposed as part of the 3GPP standardization process, but no final decision has been taken on the regulation of the usage of any particular NOMA schemes. The 3GPP technical analysis demonstrates that the high complexity of implementing algorithms for the generation and processing of signals, especially on the receiving side, is the primary challenge with the application of NOMA technology in 5G networks. Not only that but there are also novel techniques for modulation and multi-user reception, efficient algorithms for encoding and generating code, additionally, efficient strategies for integrating NOMA systems with orthogonal access technologies. This technology would be useful for a variety of uplink and downlink application scenarios in both current and future 5G and 6G systems; it would allow for multiple accesses for subscribers in a variety of settings. This led those involved in 3GPP standards to decide to delay the implementation of NOMA technology in 5G systems and instead keep exploring NOMA technology with code division, perhaps for use in novel scenarios.

## Figures and Tables

**Table 1:** A set *S* of WBE sequences from the 3GPP report.

	No. of Sequences	1	2	3	4
Sequence count down	1	−0.661 + 0.101i	−0.122 + 0.521i	0.526 − 0.428i	0.291 − 0.523i
	2	0.096 + 0.582i	−0.432 − 0.095i	0.254 − 0.323i	0.621 + 0.254i
	3	−0.432 − 0.291i	−0.583 + 0.382i	−0.391 − 0.167i	−0.591 − 0.342i
	4	−0.218 + 0.362i	0.482 + 0.241i	0.620 − 0.091i	−0.494 + 0.014i
	**No. of Sequences**	**5**	**6**	**7**	**8**
Sequence count down	1	−0.572 − 0.214i	−0.341 + 0.125i	0.421 + 0.118i	0.470 + 0.213i
	2	0.061 − 0.644i	0.367 − 0.143i	−0.024 − 0.561i	0.048 − 0.424i
	3	0.353 − 0.288i	0.651 − 0.066i	−0.450 + 0.098i	0.407 + 0.160i
	4	−0.107 − 0.461i	0.217 + 0.486i	−0.516 + 0.111i	−0.490 + 0.362i

**Table 2:** Optimizations of various parameters, and relative impact on the Gram determinant.

TSC	μ	$d_{cord}$
$\left[\begin{array}{ccc} 1 & p_{12} & \begin{array}{cc} p_{13} & p_{14} \end{array}\\ p_{12} & 1 & \begin{array}{cc} p_{23} & p_{24} \end{array}\\ \begin{array}{c} p_{13}\\ p_{14} \end{array} & \begin{array}{c} p_{23}\\ p_{24} \end{array} & \begin{array}{cc} \begin{array}{c} 1\\ p_{34} \end{array} & \begin{array}{c} p_{34}\\ 1 \end{array} \end{array} \end{array}\right]$	$\left[\begin{array}{ccc} 1 & p & \begin{array}{cc} p & p \end{array}\\ p & 1 & \begin{array}{cc} p & p \end{array}\\ \begin{array}{c} p\\ p \end{array} & \begin{array}{c} p\\ p \end{array} & \begin{array}{cc} \begin{array}{c} 1\\ p \end{array} & \begin{array}{c} p\\ 1 \end{array} \end{array} \end{array}\right]$	$\left[\begin{array}{ccc} 1 & 0 & \begin{array}{cc} p_{13} & p_{14} \end{array}\\ 0 & 1 & \begin{array}{cc} p_{23} & p_{24} \end{array}\\ \begin{array}{c} p_{13}\\ p_{14} \end{array} & \begin{array}{c} p_{23}\\ p_{24} \end{array} & \begin{array}{cc} \begin{array}{c} 1\\ 0 \end{array} & \begin{array}{c} 0\\ 1 \end{array} \end{array} \end{array}\right]$

## Data Availability

Not applicable.
